# A Phytocomplex Obtained from *Salvia officinalis* by Cell Culture Technology Effectively Controls the Grapevine Downy Mildew Pathogen *Plasmopara viticola*

**DOI:** 10.3390/plants11202675

**Published:** 2022-10-11

**Authors:** Isabella Busato, Oriana Bertaiola, Silvio Tundo, Chiara Guarnerio, Marco Lucchetta, Luca Sella, Giovanna Pressi, Francesco Favaron

**Affiliations:** 1 Department of Land, Environment, Agriculture and Forestry, University of Padova, I-35020 Legnaro, Italy; 2 Aethera Biotech s.r.l., I-36043 Camisano Vicentino, Italy; 3 Coccitech s.r.l., I-31020 San Vendemiano, Italy

**Keywords:** *Plasmopara viticola*, rosmarinic acid, *Salvia officinalis*, cell culture technology, secondary metabolites, crop protection, natural fungicide

## Abstract

The negative impact of using conventional fungicides in plant disease protection has increased the interest in safer alternatives such as plant secondary metabolites, generally having a better toxicological profile. However, cultivation conditions and plant material strongly affect the quality and quantity of secondary metabolites obtained from field grown plants, limiting the standardization needed for industrial production. Plant cell culture technology can provide highly homogeneous biomasses with specific chemical characteristics. A phytocomplex with high rosmarinic acid content (10.12% *w*/*w*) was obtained from a selected cell line of *Salvia officinalis* and was tested against the grapevine downy mildew pathogen, *Plasmopara viticola*. Grapevine leaf discs were sprayed with the phytocomplex at 5 g/L and then inoculated with *P. viticola* sporangia. Sporulation level on each disc was assessed after 7 days with an image processing software. The phytocomplex reduced by 95% the sporulation level compared to the control and was also more effective than rosmarinic acid alone, used at the same concentration found in the phytocomplex. Persistence of the phytocomplex was also assessed: when applied 5 days before inoculation, it reduced by 90% the sporulation level compared to the control. These results highlight the possibility to take advantage of cell culture techniques to produce safer pesticides with high quality standards.

## 1. Introduction

The extensive and uncontrolled use of synthetic pesticides significantly impacts human health and the environment [1]. Many countries are introducing more stringent regulations on the number and dosage of active compounds, answering public concerns. For example, the Farm2Fork strategy published by the European Commission in 2020 draws a 30-year roadmap for the transition to a sustainable food production system in Europe. As such, already by 2030, European countries will need to reduce the overall use and risk of chemical pesticides by 50% [2].

In vineyards located in temperate and humid climatic areas, fungicides are widely employed to control the grapevine downy mildew disease caused by the oomycete *Plasmopara viticola*. This pathogen, native to North America, was introduced in Europe in the 1870s with American grape cuttings [3], soon after spreading to all wine-producing countries worldwide [4].

Overwintering oospores found in leaf debris are responsible for primary infections producing sporangia that release mobile zoospores. After encystment, developing hyphae enter and colonize plant tissues through stomata. Secondary zoospores are then produced by the emerging pathogen. High humidity and mild temperatures can lead to a 75% yield reduction in untreated vineyards [5]. Because traditional *Vitis vinifera* varieties are highly susceptible to *P. viticola* and resistant ones are not favored by the market, vineyards heavily rely on synthetic fungicides and copper-based products.

In this scenario, the search for safer alternatives to traditional pesticides appears urgent. In this regard, there has been a renewed interest in natural compounds for disease protection, including secondary metabolites from plants and other organisms. These compounds generally have lower environmental persistence [6] and a better toxicological profile, with few exceptions [7]. The scientific community has identified many essential oils and plant extracts having pesticide potential [1,7,8,9]; however, only a few are now commercialized crop protection products [7]. Among the limits to using natural products in agricultural practice are the rapid degradation and volatilization of bioactive compounds in the field and the difficult standardization of the production process. Plant genetics, agronomic practices, climatic conditions, harvesting moments and extraction methods strongly affect the quality and quantity of the secondary metabolites [8]. Standardization limits can be overcome with the use of plant cell cultures.

Plant cell culture technology is a technique for growing plant cells under controlled environmental conditions. Undifferentiated plant cells are considered totipotent and have the potential to express the whole genetic machinery coded in the nucleus. Thus, they can produce the full spectrum of characteristic secondary metabolites found in mother plants [10]. Plant cells are amenable to good manufacturing practice (GMP) procedures and can be easily propagated using large volume bioreactors regardless of climate, soil or field management practices [10,11]. Moreover, in vitro cultured plant cells typically grow fast and can accumulate large amounts of uniform biomass in a short time [12,13]. Additionally, plant cell culture technology offers a reliable and robust production platform for continuous generation of contamination-free, phytochemically uniform biomass from herbal, aromatic, medicinal, and even rare and threatened plant species [14].

*Salvia officinalis* cell cultures are enriched in polyphenolic compounds [15]. While *S. officinalis* plant extracts are known for their inhibitory activity against bacterial and fungal phytopathogens [16,17], only few studies report the inhibitory activity of secondary metabolites produced by sage cell cultures. Rosmarinic acid is the most abundant polyphenol in sage cell cultures and has antioxidative and anti-inflammatory activities [18]. Ivanov et al. [19] reported rosmarinic acid inhibitory activity against bacteria and some *Candida* species. Rosmarinic acid antifungal mechanisms included reduction of mitochondrial activity, alteration in membrane integrity and slight inhibition of protease production. To the best of our knowledge, the rosmarinic acid effect against *P. viticola* has not been tested yet. Elicitors have been used to induce the production of specific secondary metabolites in plant cell cultures. In the case of *S. officinalis*, methyl jasmonate has proven effective in inducing rosmarinic acid production in shoot cultures [20].

In this work, a new phytocomplex, highly standardized in rosmarinic acid content, was obtained from a selected cell line of *S. officinalis*, and it was tested for its inhibitory effect against the grapevine downy mildew pathogen *P. viticola*.

## 2. Results

### 2.1. Salvia Officinalis Phytocomplex Was Obtained from a Stable and Selected Cell Line

A selected undifferentiated cell line of S. officinalis was obtained from leaf explants using the SO solid medium (see Section 4.1). In this medium, the S. officinalis cell line had a high growth rate and it was subcultured for at least 4 months to obtain a beige-colored stable callus culture with a friable texture and a constant growth rate. A fraction of the callus was suspended in SO liquid culture to obtain cell suspensions that were subcultured into bioreactors of increasing volumes. The content of rosmarinic acid increased by growing the cells in a final SO liquid medium with methyl jasmonate added as an elicitor. Finally, the cell culture preparation was dried to obtain the S. officinalis phytocomplex (SOP).

**Figure 1:**
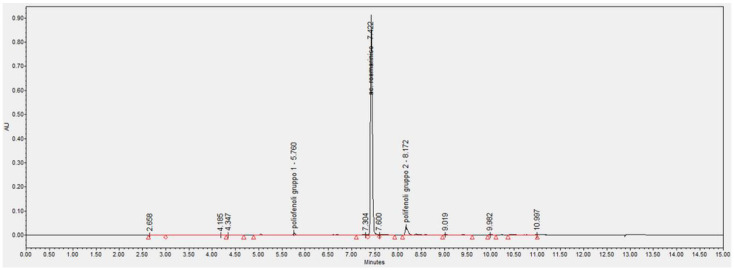
Chromatographic profile of the SOP at 330 nm of wavelength. The main peak with a retention time of 7.5 min corresponds to rosmarinic acid.

The UPLC-DAD analysis was used to estimate the content of total polyphenols and rosmarinic acid in the SOP. The chromatographic profile shows a main peak at 7.5 min corresponding to rosmarinic acid and other minor polyphenol peaks identified by their characteristic spectrum with λmax at 330 nm. Rosmarinic acid and total polyphenols were quantified using a calibration curve obtained with different concentrations of rosmarinic acid standard. The amount of total polyphenols, expressed as equivalent of rosmarinic acid, and rosmarinic acid in SOP are 11.7 ± 0.1% (*w*/*w*) and 10.12 ± 0.21% (*w*/*w*), respectively. Radical scavenging antioxidant activity (EC50 DPPH assay) was 20.94 ± 2.91 µg/mL. The chromatographic profile of the SOP extract at the wavelength of 330 nm is shown in Figure 1.

### 2.2. SOP Inhibits Plasmopara viticola Sporulation on Grapevine Leaf Discs

**Figure 2:**
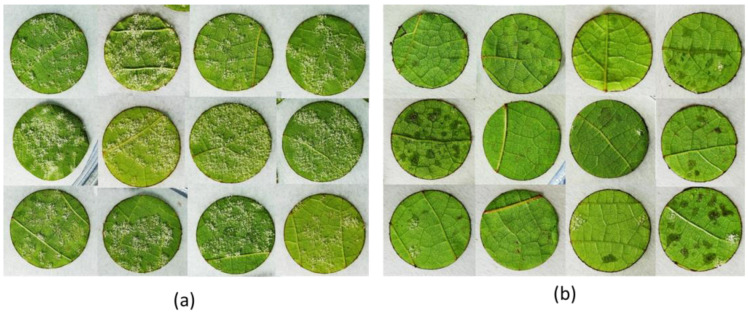
Different levels of sporulation 7 days after the inoculation with *Plasmopara viticola* on (**a**) water-treated discs (control); (**b**) *Salvia officinalis* phytocomplex (SOP)-treated discs.

The effect of SOP in preventing *P. viticola* infection was determined by analyzing the sporulation level on grapevine leaf discs inoculated with the pathogen sporangia. From preliminary experiments, no appreciable effect was observed with SOP at 1 g/L (not shown); therefore, the concentration of SOP was increased to 5 g/L. The SOP treatment at 5 g/L was compared to a treatment with 0.5 g/L rosmarinic acid, i.e., the quantity contained in 5 g/L SOP, and to a deionized water control. After spraying, the discs were allowed to dry and then were inoculated with *P. viticola* sporangia. After incubation for 7 days in the dark at 25 °C, different sporulation levels on the leaf discs were visible among treatments (Figure 2).

**Figure 3:**
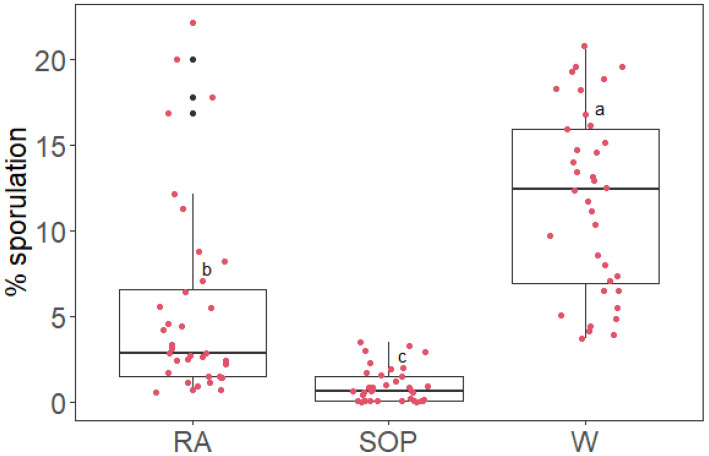
Boxplot of the percent ratio between the sporulating area and the total disc area (% sporulation) on grapevine leaf discs treated with rosmarinic acid 0.5 g/L (RA), *Salvia officinalis* phytocomplex solution 5 g/L (SOP), or deionized water (W), inoculated with *P. viticola* sporangia. Different letters indicate significant differences between mean log (% sporulation + 1) based on multiple comparisons analysis with Bonferroni correction (level of significance = 0.05). Raw data values are overlaid to the boxplot as red dots, with added horizontal jitter for visibility.

Non-susceptible leaves were identified, and discs originating from those leaves were not considered in the following steps. Non-susceptible leaves were 6 out of 10 in the first repetition of the experiment and 3 out of 10 in the second and third repetition. Sporulation levels on the discs were measured with an image processing software and reported as the percent ratio between the sporulating area and the total disc area (% sporulation). Multiple comparison analysis confirmed that the sporulation level on SOP-treated discs was significantly lower (−95%) than that of the water treatment (*p* < 0.001). Rosmarinic acid showed some level of inhibitory activity (−77%), but it was not as effective as SOP (*p* < 0.001, Figure 3).

### 2.3. SOP Effect Persists on Grapevine Leaves

**Figure 4:**
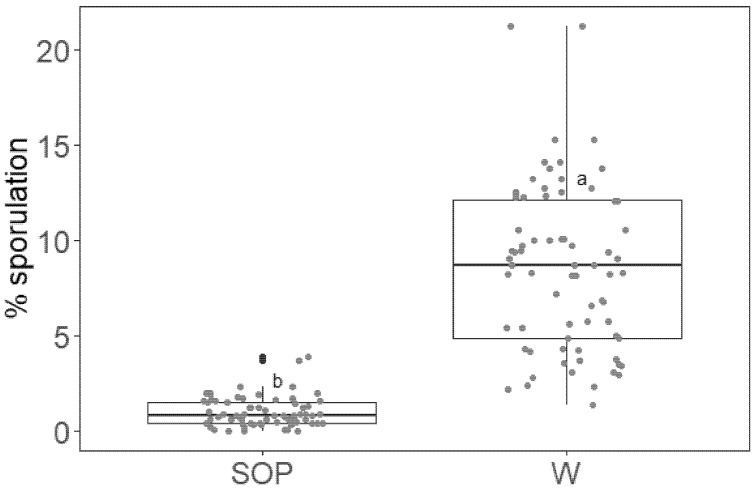
Boxplot of the percent ratio between the sporulating area and the total disc area (% sporulation) on grapevine leaf discs treated with *Salvia officinalis* phytocomplex solution 5 g/L (SOP) or deionized water (W) and inoculated 5 days later with *P. viticola* sporangia. Data from three repetitions of the experiment were analyzed together. Different letters indicate significant differences between mean log (%sporulation + 1) based on multiple comparisons analysis with Bonferroni correction (level of significance = 0.05). Raw data values are overlaid to the boxplot as red dots, with added horizontal jitter for visibility.

Grapevine leaves were sprayed with either SOP 5 g/L or water as negative control. Five days later, the treated leaves were detached from the plants and cut to obtain discs that were then inoculated with *P. viticola* sporangia. After a 7-day incubation in the dark, sporulation levels were assessed as described above. Data from three repetitions of the experiment were analyzed. No discs were excluded from the analysis because all leaves used in the experiment were susceptible. A significant 90% reduction in sporulation level was observed on SOP-treated leaf discs compared to the water treatment (Figure 4).

## 3. Discussion

Although plant cell culture technology has been extensively applied for the production of food additives, cosmetic ingredients and pharmaceuticals [21], its potential in agrochemical production has not been explored yet. A stable *Salvia officinalis* cell line was herein induced with methyl jasmonate to obtain a phytocomplex characterized by a high polyphenolic content (11.7% *w*/*w*), with rosmarinic acid as its main component (about 10% *w*/*w*). This result is of particular interest because *S. officinalis* leaves usually have a rosmarinic acid content of about 4% dry weight [22,23], and *S. officinalis* cell cultures naturally produce rosmarinic acid with a concentration up to 5% dry weight [15,20]. An extensive literature describes rosmarinic acid antioxidative and anti-inflammatory activities [18]. The high polyphenolic content of the SOP results in particularly high antioxidant activity (EC50 20.94 µg/mL) compared to other similar *S. officinalis* extracts, such as those described in Grzegorczyk et al. [24], whose extract from *S. officinalis* cell suspension culture had an EC50 at about 34 µg/mL. Inhibitory activities against bacteria and fungi have also been reported [25]. In our study, the SOP treatment at 5 g/L reduced *P. viticola* sporulation on grapevine leaves by 95%. Rosmarinic acid content in the 5 g/L SOP treatment is 0.5 g/L, comparable to the MICs reported against *Candida spp.* and bacterial species, ranging from 0.1 to 1 g/L [19]. Typical *S. officinalis* preparations used in antimicrobial tests include essential oils obtained from hydrodistillation or organic solvent extractions from plant material, primarily leaves [16,17,26]. Pinto et al. [16] reported the inhibitory effect of *S. officinalis* essential oil on five *Candida* species, five different dermatophytes and four fungal phytopathogens, namely, *Aspergillus flavus*, *Penicillium italicum*, *Cladosporium cladosporioides* and *Fusarium moniliforme*. Dagostin et al. [17] observed a relevant inhibitory activity of an ethanolic extract of *S. officinalis* against *P. viticola*. These preparations are enriched in hydrophobic terpenes and terpenoids such as borneol, camphor, thujone, humulene, pinene, cineole, to which the antimicrobial effect is attributed [16,26,27]. The SOP presented in this paper does not likely contain such compounds because the phytocomplex is obtained from undifferentiated cells [28]. Essential oils are produced in the plant by structures having a high level of differentiation, such as oil cells, secretory glands or glandular trichomes [29]. Instead, phenolic compounds are the main secondary metabolites synthesized in suspended cell cultures, and their amount can be regulated with the application of hormones or elicitors [30], such as methyl jasmonate used in this work. The inhibitory effect of SOP treatment on *P. viticola* sporulation was more pronounced than only rosmarinic acid treatment alone, which resulted in a 77% sporulation reduction. A synergistic effect between the numerous components of the phytocomplex can explain the higher efficacy of the SOP preparation compared to an equal amount of rosmarinic acid alone. Rosmarinic acid could be more effective in the SOP due to the simultaneous presence of other molecules that could enhance its bioavailability, improve its permeability into the pathogen cells and inhibit the pathogen detoxification systems (for a review on synergism in natural product extracts, see [31]). Moreover, other minor components in the phytocomplex could also have inhibitory activity. Only bioassay-guided fractionation analyses and metabolomic approaches could identify the active components and the synergies in a complex mixture such as SOP [31]. The efficacy of SOP against *P. viticola* was also maintained when the pathogen inoculation was delayed for 5 days after the application of SOP on the leaves: this is a promising preliminary result on the applicability of this SOP for plant protection purposes. Generally, the use of natural compounds in disease management is limited by their low persistence, which results in more frequent applications and higher management costs for the final user. SOP degradation rate in the environment and persistence on the treated plant need to be verified in field conditions. Curative effect was also tested by first inoculating the leaf discs with fresh *P. viticola* sporangia and then spraying the treatments 3 days later, but no difference was observed between the sporulation level on the control and on the SOP treated discs (data not shown).

The production process for the SOP was scaled up by Aethera Biotech company to produce 500 L/day of suspension culture using bioreactors. Chemical stability of the phytocomplex was verified up to 30 months with standard procedures.

Overall, these results open the possibility to produce highly homogenous biomasses from this *S. officinalis* cell line, with an increased content of secondary metabolites, to develop a safer crop protection product for preventive control of grapevine downy mildew.

## 4. Materials and Methods

### 4.1. Salvia Officinalis Cell Culture

Plants of *S. officinalis* L. were bought and certified from a plant nursery (Le Georgiche, Brescia, Italy) and used as starting material to generate a cell culture. The botanical species was confirmed through molecular biology analysis (DNA fingerprint) performed in collaboration with Padano Technology Park, Lodi, Italy [32]. Young leaves and sprouts were sterilized with 70% (*v*/*v*) ethanol (Honeywell) aqueous solution and, subsequently, with 5% (*v*/*v*) sodium hypochlorite solution (6–14% active chlorine, Merck, Milano, Italy) and 0.1% (*v*/*v*) Tween 20 (Merck, Milano, Italy). The plant tissue was then cut into minute fragments (explants) and deposited in Petri dishes containing agarized Gamborg B5 nutrient medium [33] with different combinations and concentrations of plant growth regulators and incubated at 25 °C in the dark.

The highest rate of callus growth was observed using the Gamborg B5 medium containing 0.8% (*w*/*v*) of Plant Agar (Duchefa Biochemie, Haarlem, The Netherlands) and supplemented with 20 g/L sucrose (Sudzucker, Mannheim, Germany), 1 mg/L of naphthalene acetic acid (NAA, Duchefa), 0.5 mg/L of Kinetin (K, Duchefa) and adjusted to pH 6.5. Calli grown on this medium (designated SO solid medium) were subcultured every 14 days for at least 4 months until they became friable, homogeneous and with a constant growth rate (*S. officinalis* stable cell line).

Cell suspension cultures were generated by transferring 15% (*w*/*v*) of selected callus into 250 mL Erlenmeyer flasks containing Gamborg B5 medium supplemented with 20 g/L sucrose, 1 mg/L of NAA, 0.5 mg/L of K, and adjusted to pH 6.5 before autoclaving. The cells were grown on this medium (designated SO liquid medium) in the dark at 25 °C on a rotary shaker (120 rpm) and subcultured in a fresh liquid medium every 7 days. An aliquot (6% *v*/*v*) of cell suspension was transferred and adapted to grow in bioreactors of progressively increasing volumes (3 l, 5 l and 15 l). To increase the content of rosmarinic acid and total polyphenols, after 7 days of fermentation in the SO liquid medium, the cell suspension was transferred to a final Gamborg B5 medium at pH 6.5 supplemented with 40 g/L of sucrose, 2 mg/L of NAA, 1 mg/L of K, and 15 mg/L of methyl jasmonate (Merck, Milano, Italy) as an abiotic elicitor (SO final liquid medium). The methyl jasmonate stock solution was prepared by dissolving 400 mg of methyl jasmonate in 10 mL of 70% (*v*/*v*) ethanol in water. The cell culture was grown for a culture cycle of 14 days in the dark at 25 °C in a bioreactor of 15 l volume containing 10 l of SO final liquid medium.

### 4.2. Phytocomplex Preparation from Salvia officinalis Cell Culture

After 14 days of growth in the SO final liquid medium, the *S. officinalis* cells were collected by filtering the suspensions with a 50 µm mesh membrane (nylon mesh, Elko Filtering Co., LLC, Switzerland). The cells were washed with a double volume of saline solution (0.9% *w*/*v* NaCl in sterile water), and citric acid was added until reaching a pH of about 3.0. The mixture was homogenized with an Ultraturrax homogenizer at 15,000 rpm for 20 min and dried using a Mini Spray Dryer (BUCHI-B290, Cornaredo, Italy) to obtain a powder of *S. officinalis* phytocomplex (SOP).

. Antioxidant radical scavenging activity of the SOP was determined according to the 1,1-diphenyl-2-picrylhydrazyl (DPPH) assay, as reported by Rahman et al. [34]. It was expressed as EC50, which corresponds to SOP concentration that gives 50% scavenging activity of the DPPH radical.

### 4.3. Quantification of Rosmarinic Acid in the S. officinalis Phytocomplex by UPLC-DAD Analysis

One hundred milligrams of SOP powder was weighed into a 15 mL test tube and 30 volumes of ethanol:water 60:40 (*v*/*v*) were added. The suspension was vortexed for 30 s, sonicated for 15 min in an ice bath, and finally centrifuged at 4000 rpm for 15 min at 6 °C. The supernatant was collected, diluted 1:10 (first 1:5 in ethanol and then 1:2 in water) and filtered by a 0.22 μm membrane before being loaded into the UPLC system. Five independent extracts of SOP were analyzed by a chromatographic UPLC-DAD system (Waters, Sesto San Giovanni, Italy) equipped with an Acquity UPLC BEH C18 1.7 μm column (size 2.1 × 100 mm), coupled with an Acquity UPLC BEH C18 1.7 μm VanGuard Pre-Column (size 2.1 × 5 mm). The chromatographic columns were eluted for 1 min with 99% of solvent A (0.1% formic acid in water, *v*/*v*) and 1% of solvent B (100% acetonitrile), for 10 min with a linear gradient from 1 to 40% of solvent B, and 1 min from 40 to 100% of solvent B. The run was performed at 30 °C at a constant flow rate of 0.350 mL/min, and the absorbance was read at 330 nm wavelength.

Rosmarinic acid was identified and quantified by the external standard method using a calibration curve performed with a commercial rosmarinic acid standard (CAS 20283-92-5, purity ≥ 98%, code R4033, Merck, Milan, Italy) and the Empower 3 software (Waters). To establish the calibration curve, known concentrations of the standard solutions were plotted against the peak areas measured at 330 nm. The linear regression equation was calculated via the least square method. This method showed excellent linearity at concentrations from 0.2 to 160.3 µg/mL; the linear correlation coefficient (R^2^) was 0.9999.

Total polyphenol content, including rosmarinic acid and other minor components, was estimated using the sum of all peak areas at 330 nm and the rosmarinic acid calibration curve.

### 4.4. Plant Material

*Vitis vinifera* cv. Glera shoots were potted and grown in greenhouse conditions. In February, the plants were pruned, leaving 4 buds per plant. For the inoculation experiments, only the third to sixth unfolded leaves of the shoot, counted from the apex of the shoot, were used.

### 4.5. SOP and Rosmarinic Acid Preparations

SOP was suspended at 10 g/L in deionized water, vortexed vigorously and centrifuged at 8000 g for 15 min. The supernatant was filter-sterilized by 0.20 μm membrane (Sarstedt, Nümbrecht, Germany) and stored at 4 °C as a SOP stock solution until use. Rosmarinic acid (code R4033, Merck, Milan, Italy) was dissolved in pure ethanol to obtain a stock solution of 200 g/L.

### 4.6. Leaf Inoculation with Plasmopara viticola

*P. viticola* sporangia were obtained from sporulating leaves of *V. vinifera* cv. Glera from a vineyard in Nervesa della Battaglia (Veneto region, Italy). The inoculum was collected by washing the leaves with sterile deionized cold water, and the sporangia suspension was then sprayed on the abaxial surface of fresh grapevine leaves. The inoculated leaves were incubated in a humid chamber at 25 °C in the dark for 7 days to produce fresh inoculum. The inoculation procedure was repeated on new fresh leaves to obtain the necessary inoculum for SOP assays. Once collected, the inoculum necessary for SOP assays was adjusted to 5 × 10^5^ sporangia/mL and immediately sprayed. During the cold season, sporangia vitality was preserved by storing air-dried sporulating leaves at −80 °C.

### 4.7. SOP Inhibitory Assay against Plasmopara viticola Infection

The inhibitory activity of SOP at 5 g/L against *P. viticola* infection was compared to rosmarinic acid at 0.5 g/L. SOP and rosmarinic acid were dissolved in water, and the solutions had a pH of about 3.0. The control treatment was performed with deionized water (W).

**Figure 5:**
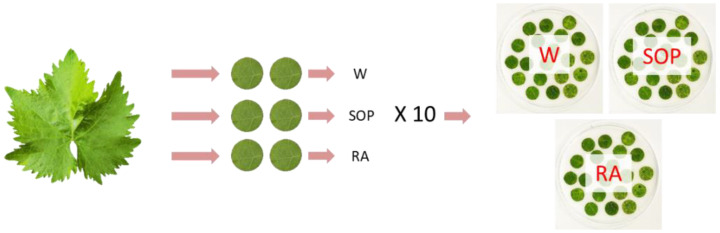
Experimental setup for inhibitory assay. From each leaf, 6 discs were obtained and assigned in equal number (two) to each of the three treatments: deionized sterile water (W), *Salvia officinalis* phytocomplex solution 5 g/L (SOP), rosmarinic acid 0.5 g/L (RA). A total of 10 leaves were used, obtaining three plates with 20 discs in each. The experiment was repeated 3 times.

Three Petri plates with wet filter paper bottom lining were prepared, one for each treatment. Six discs (1.7 cm diameter) per leaf were excised with a cork borer from 10 freshly harvested grapevine leaves and randomly distributed, abaxial side up, among the plates (Figure 5) to obtain 20 leaf discs per plate, for a total of 60 discs. The discs were given codes based on the plant and leaf of origin.

Each plate was sprayed with 4 mL of the respective treatment and allowed to dry. Subsequently, the inoculum of *P. viticola* was sprayed on the surface of the leaf discs. The plates were closed and incubated at 25 °C in the dark. Disease severity was assessed 7 days after inoculation. Discs originating from non-susceptible leaves that did not produce any sporulation, neither in the control nor in the treated samples, were not considered in the subsequent analysis.

**Figure 6:**
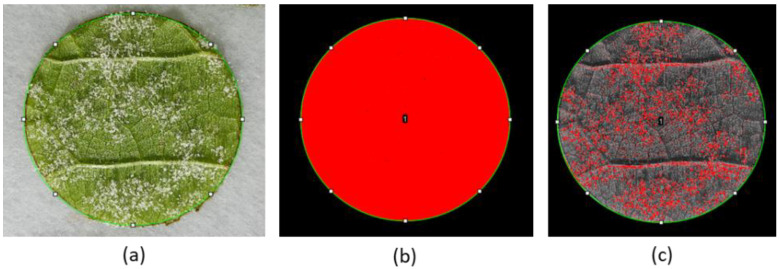
Image processing: (**a**) picture of a sporulating leaf disc, 7 days after inoculation with *Plasmopara viticola* sporangia; (**b**) the pixels of the entire disc are selected (red) and measured; (**c**) with the color threshold function, only the pixels of the white sporangia are selected (red) and measured.

Disease severity assessment was conducted by processing the picture of each disc with Fiji [35] and setting different color thresholds to distinguish the pixels of the area covered by the sporangia (Figure 6) [36].

The disease severity (% sporulating area) was then calculated as the ratio between the pixels of the sporangia and the pixels of the entire disc, expressed in percent. The experiment was repeated 3 times, using a total of 30 leaves and 180 leaf discs.

### 4.8. Persistence of SOP Treatment

The persistence of SOP activity was assessed on 4 plants (two per treatment) randomly assigned to each of two different treatments: deionized sterile water (control) or SOP (5 g/L). Three leaves from every plant were sprayed with the corresponding treatment on the abaxial surface. Because treatments could have a systemic effect on the plant and thus affect the disease susceptibility of untreated leaves, each plant was sprayed with only one treatment. After 5 days, the treated leaves were detached, and 4 discs from each leaf were excised and transferred, abaxial side up, to the corresponding Petri plate with wet filter paper bottom lining. Subsequently, the inoculum was sprayed simultaneously on all the plates. The plates were incubated at 25 °C in the dark. Disease severity expressed as sporulation area was assessed as described above. The experiment was repeated 3 times, using 12 plants, 36 leaves and 144 leaf discs.

### 4.9. Statistical Analysis

All statistical analyses were conducted with the R software [37]. Right skewed data distributions were normalized with the function log(x + 1). A mixed-effects ANOVA model was then applied, considering treatment and replica as fixed effects, and leaf as a random effect. In order to assess the goodness-of-fit of the model, normality of residuals and autocorrelation was tested. Significant differences between treatments were identified with multiple comparisons using Bonferroni correction.

Data were plotted on boxplots: boxes range from the first quartile (Q1) to the third quartile (Q3), and the line across the box represents the median. Whiskers extend from Q3 − 1.5 (Q3 − Q1) to Q3 + 1.5 (Q3 − Q1). Outliers are represented by black dots.

## Data Availability

Not applicable.
